# Novel Insights into Solution Electrospinning for Nanofibers

**DOI:** 10.1021/acs.macromol.5c00703

**Published:** 2025-06-17

**Authors:** Chi Wang, Pin-Hsien Lu, Yin-Chuan Kuo, Chih-Hsien Kuo, Hsin-Yi Lai, Shao-Hua Wu, Takeji Hashimoto

**Affiliations:** † Department of Chemical Engineering, 34912 National Cheng Kung University, Tainan 701, Taiwan 70101, ROC; ‡ 12918 Kyoto University, Kyoto 606-8501, Japan

## Abstract

To understand how nanofibers are formed by solution electrospinning, an in situ observation of the fluid flow is essential. In this study, the flow behavior of charged fluids from the Taylor cone, straight jet part, until whipping (or spiral) jet part along the spinline is explored via particle image velocimetry, light scattering, and high-speed videography, respectively. The results of light scattering reveal that the stretching rate in the straight jet exceeds the intrinsic rates of polymer relaxation in the fluid, derived from the dynamic rheological measurement, supporting the hypothesis of flow-induced phase separation and the resultant evolution of the dissipative structures, the so-called “string” structures, in the straight jet section. Using liquid nitrogen to collect the straight jet followed by freeze-drying, assembled strings with various widths are validated. Moreover, dynamic vortex flow is observed in the Taylor cone using particle image velocimetry, likely generating a swirling flow in the cone apex. The vorticity of the swirl is increased after passing the cone-jet transition zone, at which the electric field is the highest. Thereafter, the enhanced swirl gradually decays (or releases its imposed torsion) during its propagation along the straight jet via a jet twist. The straight jet with internal swirl is considered as the precursor of the spiral jet, given that the preimposed torsion in the straight jet is not completely relaxed at the straight jet end. Using high-speed videography, a transition of the handedness of the spiral jet, rotating either clockwise or counterclockwise, is repeatedly observed in a single spinning line, suggesting the intermittent entry of the swirl with different handedness in the cone apex. Thus, the downstream spiral jet is relevant to the upstream entry flow at the cone apex; this phenomenon resembles the classic extrusion instability. Helical fibers (or coils) are observed on the ground collector, as the residual torsion in the spiral jet is not fully released after solvent evaporation in the spinning line of the spun fiber. Our work shows that important external flow fields are applied to semidilute solutions through the electrospinning process, which self-organizes nanofibers as ordered structures from dissipative structures through thermal concentration fluctuations along the spinning line, starting from the needle tip to the whipping jet.

## Graphical abstract

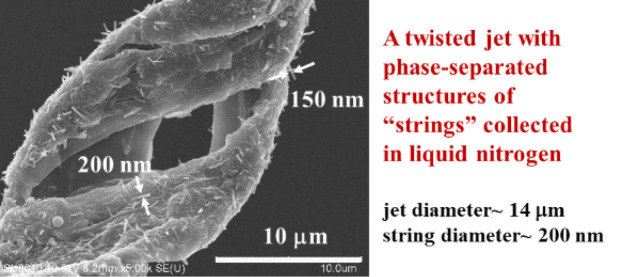

## 1 Introduction

Electrospinning has emerged as a frequently deployed technique for preparing polymer nanofibers for practical applications in numerous fields, such as filtration, biomaterial scaffolds, and composites, as the nanofiber mats exhibit high specific surface areas and strength.^1–5^ However, the fiber formation mechanism has not been sufficiently clarified, despite the challenges accompanying the in situ observations of the whole process, from the Taylor cone to the jet whipping region. These challenges are attributable to the extremely short (several to tens of milliseconds) and extremely small sizes of the flying jet (diameters of several to tens of microns). It is long believed that the electrospinning jet is subjected to very high stretching rates, which generate high chain orientations in the as-spun fibers following solvent removal. However, our recent study^6,7^ was the first to quantitatively investigate the stretching rates of the fluid elements in the straight jet as well as in the bending (or spiral) jet. Moreover, the rheological properties, e.g., the viscosity and relaxation rate, of the polymer solutions employed for electrospinning are key to determining the properties of the final fiber.^6,8^ Additionally, flow-induced phase separation is likely to occur in the spinline if the electrical-stress-induced stretching rate exceeds the intrinsic relaxation rate of the polymer chains in the solution.^6,9,10^

In our previous study,^11^ we electrospun poly­(vinyl alcohol) (PVA) with a hydrolysis degree of 99% to demonstrate the existence of phase-separated structures embedded in the straight jets that were feasibly collected in a nonsolvent reservoir to induce the freezing of the internal structure of the straight jet for subsequent examinations using optical and electron microscopies.^11^ Based on these ex-situ observations, we proposed a novel fiber formation mechanism^12^ wherein flow-induced phase separation would occur to produce the dissipative structures of strings in the spinline, which are the precursors of the as-spun fibers on the grounded collector. The physics underlying the evolution of the dissipative structures is that the evolution occurs at a dynamic balance between the dissipation of the mechanical energy stored in the stretched solution and the thermodynamic free energy required to evolve the internal structures of the jet, driven by the flow-induced thermodynamic instability inherent to the stretched solution under the elongational flow fields. Accordingly, the evolution of various dissipative structures is anticipated to occur in a cascade as a function of the distance from the needle tip to the grounded collector along the spinline.^12^ Our proposed mechanism is at variance with the existing knowledge that assumes the one-phase polymer solution is continuously stretched to reduce the jet diameter along the spinline; this process is accompanied by the increased polymer concentration attributed to solvent evaporation from the jet until the dried nanofibers are formed and deposited on the collector.

In this study, two types of PVA with similar average molecular weights but different degrees of hydrolysis were used to prepare aqueous solutions for electrospinning. To directly explore the internal structures of the straight jet, a liquid nitrogen (LN_2_) bath was used to collect the flowing jet (Figure S1) to induce the abrupt freezing of the straight jet, which may have already evolved the dissipative structures via the elongational-flow-induced phase separation. In addition, we found the existence of the dynamic vortex flow in the Taylor cone and the intermittent change in the rotational direction of the spiral jet in the bending (or spiral) jet part along the spinline. A plausible coupled relation is proposed between the upstream vortex flow and the downstream spiral jet.

## 2 Experimental Section

### 2.1 Solution Properties

Two different PVA samples with similar average molecular weight of 166 000 g/mol, but different degrees of hydrolysis (i.e., 88% and 98%) were obtained from Sigma-Aldrich Co.; they are denoted as PVA88 and PVA98, respectively. Aqueous solutions with 7 wt % PVA concentration were prepared at 95 °C under constant stirring for several hours to ensure concentration homogeneity of the solution. The linear viscoelastic properties of the solutions at different temperatures (15–30 °C) were determined using a rheometer (ARES) with a cup-and-bob feature. Small-amplitude oscillatory shear mode was used to obtain the storage modulus $G^{\prime }$ and loss modulus $G^{\prime \prime }$ over a range of angular frequencies (ω), from which the zero-shear viscosity η_0_ and recoverable shear compliance $J_{s}^{o}$ were determined from the *G’*(ω) and $G^{\prime \prime }\left(\omega \right)$ data at low frequencies in the terminal flow region: $\eta_{0}=\lim_{\omega \to 0}G^{\prime \prime }\left(\omega \right)/\omega$ , and $J_{s}^{o}=\left(1/\eta_{0}^{2}\right)\lim_{\omega \to 0}G^{\prime }\left(\omega \right)/\omega^{2}$ .

Surface tension γ and conductivity κ of the prepared 7 wt % PVA κ solutions were measured at 25 °C using a Kyowa surface tension meter (DY-300) and a Thermo conductivity meter (Cyberscan PC510). The measured γ was 47.2 ± 0.1 mN/m and 50.9 ± 0.2 mN/m for the PVA88 solution and PVA98 solution, respectively, whereas κ was 690 ± 5 μS/cm and 1707 ± 5 μS/cm for the PVA88 solution and PVA98 solution. Thus, the 7 wt % PVA98 solution possessed higher γ and larger κ than the PVA88 solution because of the greater degree of hydrolysis.

### 2.2 Electrospinning and Light Scattering

The homogeneous polymer solution at 25 °C was delivered by a syringe pump at a flow rate (*Q*) of 0.2 mL/h through the PTFE tubing into stainless steel needles with an outer diameter of 1.47 mm and an inner diameter of 1.07 mm. High electrical voltage was applied to the needle spinneret. To construct a needle-plate electrode configuration, a steel plate (30 × 30 cm^2^) was used to collect electrospun fibers at a tip-to-collector distance (*H*) of 21 cm below the needle tip. To achieve a stable cone-jet electrospinning mode, the voltage range was determined to be 8.0–10.1 kV for the PVA88 solution and 9.4–10.2 kV for the PVA98 solution. Thus, a common voltage (*V*) of 9.5 kV was selected to electrospin both solutions of PVA for comparison. The dynamics of the electrospinning jet was examined by using a high-speed video (Fastec IL4) equipped with a telescope lens to keep a distance from the charged flowing jet.^7^ The current carried by the PVA fibers depositing on the collector was also measured by a digital multimeter (Keysight Co., model: 34465A) during electrospinning of the PVA88 solution and PVA98 solution, which were found to be 415 ± 5 nA and 393 ± 11 nA, respectively.

The jet diameter and jet interior were analyzed using the light scattering technique with a polarized He–Ne laser equipped with a pinhole of 0.4 mm diameter as the light source. The laser light position on the straight jet was carefully controlled by two moving stages connected orthogonally (Figure S2). Using a Neo 5.5 CCD (Andor Solis Co.), the scattering patterns of the liquid jet at different distances *z* from the needle tip (*z* = 0) along the spinline were collected on a screen behind the jet. A large, polarized sheet was placed in front of the screen to act as an analyzer to obtain the V_V_ scattering pattern. The data acquisition time required to capture appropriate pattern images for analysis was 1 ms. The intensity profile along the equator was plotted as a function of the magnitude of the scattering vector *q* [= (4π/λ)­sin­(θ/2), where θ and λ are the scattering angle and the wavelength of the incident beam in the solution, respectively]. Similar setup has been successfully applied to study the electrospinning of poly­(*N*-isopropylacrylamide) (PNIPAM) solutions in dimethylformamide (DMF) to obtain the jet diameter, and some details were provided elsewhere.^6^ The main schemes to derive the jet diameter are listed as follows. The laser beam diameter is about 0.4 mm to cover the straight jet at a given *z* with a diameter of several μm, which decreases with *z.* Within the irradiated volume of laser light beam, the jet at a given *z* is taken as a long cylinder so that the scattering intensity from the jet as a whole can be described by the Mie scattering.^6^ According to the Mie scattering under the V_V_ polarization, the intensity profile *I*(*q*) depends solely on two parameters only, i.e., the jet diameter (*d*_j_) and the refractive index (*n*_av_) of the solution jet. Experimentally, successive scattering peaks are normally observed in a wide *q* range, and the position of the strong first peak (*q*_m1_) is used to determine the jet diameter at a given *n*_av_.

### 2.3 Particle Image Velocimetry

Figure S3 shows the schematics for particle image velocimetry used to trace the fluid flow in the Taylor cone. Green laser source (300 mW) coupled with a Powell lens (Laserline Optics, Canada) with a fan angle of 5° was used to produce a fan-shaped incident laser beam so that the incoming laser beam spreads into a fan with thickness of 1.5 mm and an apex angle of 5° in the horizontal plane to irradiate the Taylor cone. The wavelength of the laser source was 0.532 μm. The direction of the laser sheet was precisely adjusted using two orthogonal stepper motors. The viewing angle (Φ) of the CCD with respect to the laser beam direction was about 135°. Hollow glass beads with an average diameter of 6 μm and a density of 1.1 g/cm^3^ were used, and the bead concentration in the polymer solution was 85 ppm.

### 2.4 Morphological Observations of Jets and Fibers

The flying jets during electrospinning were collected in liquid nitrogen (Figure S1). Freeze-drying was subsequently carried out to remove the solvent. After solvent removal, the morphologies of the collected jets were examined by using a scanning electron microscope (SEM, Hitachi SU8010) as well as a transmission electron microscope (TEM, Jeol JEM1400). Nonsolvent 1-propanol was also applied to collect the flying jet to solidify the developed structures in the electrospinning jet.^12^ Ex-situ observations of the collected jets were carried out by using a polarized optical microscope (POM, Leica DMLP), SEM, and TEM.

## 3 Results and Discussion

### 3.1 Viscosity and Relaxation Rate of Polymer Solutions

The frequency dependence of the rheological properties *G”* and *G’* of the 7 wt % PVA88 solution at different temperatures is shown in Figure 1. The low-frequency terminal flow region can be reached because *G’* varies as ω^2.0^ and *G”* varies as ω^1.0^, regardless of the solution temperature. The corresponding values of η_0_ and $J_{s}^{o}$ were derived from the intercepts at the given temperature; afterward, the terminal relaxation time τ_d_ was subsequently calculated from τ_d_= η_0_ $J_{s}^{o}$ . The determined values of η_0_, τ_d_, and relaxation rate (τ_d_^–1^) are shown in Table 1. As the solution temperature is increased, both η_0_ and τ_d_ are decreased. At a given temperature, the PVA88 solution possesses a larger shear viscosity and a longer relaxation time, τ_d_, than the viscosity and the relaxation time of the PVA98 solution.

**Figure 1:**
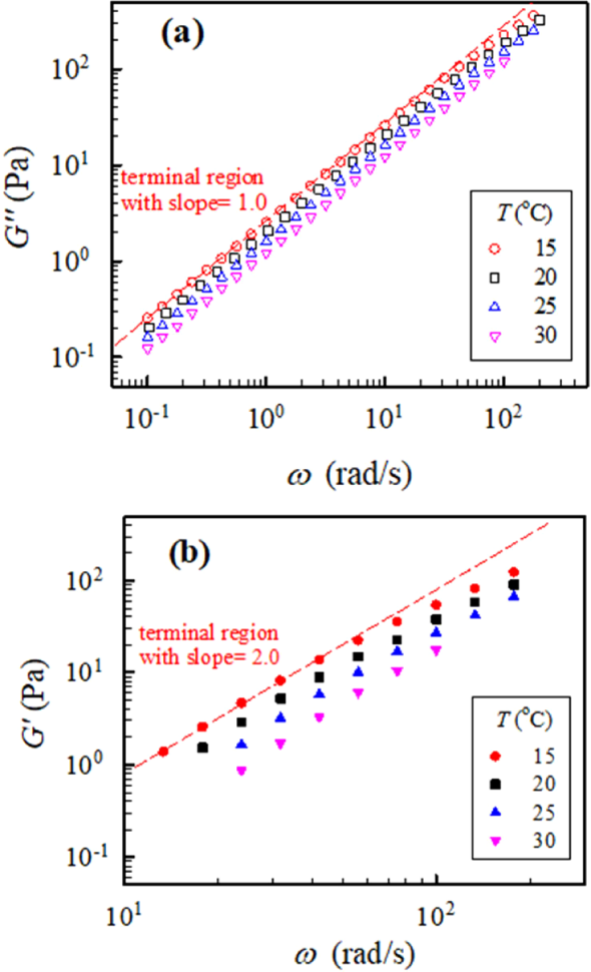
(a) Dynamic loss modulus *G”* and (b) dynamic storage modulus *G’* of the 7 wt % PVA88 aqueous solution as a function of applied frequency ω at different temperatures.

**Table 1:** Temperature Dependence of the Zero-Shear Viscosity *η*_0_, Terminal Relaxation time *τ*_d_ and Relaxation Rate *τ*_d_^–1^ of the 7 wt% PVA/Water Solutions

	PVA88 solution			PVA98 solution		
*T* (°C)	η_0_ (cP)	τ_d_ (ms)	τ_d_^–1^ (s^–1^)	η_0_ (cP)	τ_d_ (ms)	τ_d_^–1^ (s^–1^)
15	2560	3.2	313	2150	2.1	476
20	2020	2.6	385	1160	1.5	667
25	1620	2.0	500	910	1.2	833
30	1250	1.5	667	730	1.0	1000

The structure of our discussions on electrospinning is organized as follows. In Section 3.2, direct evidence of the phase-separated structures of strings is first provided. Theoretical discussions of the flow-induced phase separation are given in Section 3.3, and the flow field in the Taylor cone is given in Section 3.4. In Sections 3.5 and 3.6, the vortex flow in the Taylor cone is correlated to the rotation of the spiral jet as well as the helical fibers on the collector. Finally, the hydrolysis effects of PVA on the jet and fibers are provided in Section 3.7.

### 3.2 Evidence of Flow-Induced Phase Separation in the Straight Jet

Figure 2 shows the SEM images of the LN_2_-collected PVA/H_2_O jet after being freeze-dried to remove the solvent. Several notable insights can be drawn: (i) direct evidence of the flow-induced phase separation in the jet demonstrates the various assemblies of string structures with different widths existing as the internal dissipative structures developed in the phase-separated straight jet; (ii) the determination of the smallest width of the strings (150–300 nm; Figure 2c,e), corresponding to the diameters of the PVA fibers collected on the grounded collector (Figure S4), may indicate that the so-called “electrospun fibers” on the collector originated from the splitting of the internal string structures developed in the phase-separated jet from the jet in the spinline during electrospinning;^12^ and (iii) the straight jet may be twisted (Figure 2d), indicating that a rotating flow field exists upstream in the Taylor cone, likely in the entrance region of the cone apex where the convergent flow occurs (as will be detailed in Figure 5). In other words, the fluid elements, before entering the straight-jet region, already exhibited the rotating-velocity component, e.g., a swirling or vortex flow.^13–15^

**Figure 2:**
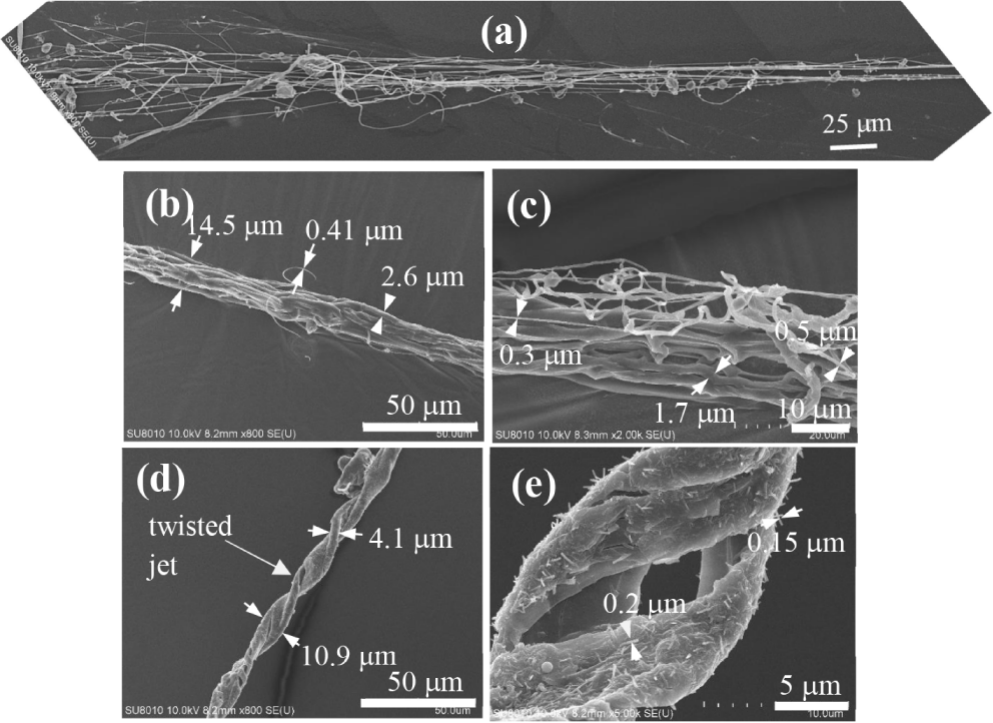
Direct evidence of flow-induced phase separation in the straight jet. The SEM images were obtained from the PVA88/H_2_O jet after the straight jet being collected into LN_2_ bath, followed by freeze-drying of the frozen jet. The following three typical jet morphologies were observed: (a) rapidly frozen jet, which is spread out from the flow-induced phase-separated straight-jet solution into structures of strings with various widths; the flow is expected to be from the right to left directions, (b) untwisted jet segment (diameter = ∼15 μm) comprising moderately twisted string bundles, (c) enlarged portion of (b) to disclose the strings with different widths, (d) twisted jet segment (diameter = ∼ 11 μm), and (e) enlarged portion of (d) to disclose the tiny fibers with diameters of 150–200 nm.

In the straight-jet regime, which lacks a rotating electric field, the twisted jet may relax its imposed torsion, inducing the clockwise (or counterclockwise) rotation of the straight jet around the jet axis. Notably, Bellan et al.^16^ observed the transverse oscillatory movement of the tracer particle in the straight jet, potentially accounting for the downstream bending instability (i.e., the whipping jet). Moreover, the straight jet was axially stretched by the electric force under the axial electric field along the jet direction to reduce the jet diameter, thereby increasing its velocity. As the straight jet gradually thins out, the air drag plays a dominant role in resisting the jet flow and compressing (or buckling) the straight jet under a critical condition^6^ to subsequently initiate the jet-bending instability for lateral jet motion. It is observed that the bending jet exhibits a spiral motion, whose origin could be attributable to the relaxation of the residual torsion (Section 3.6).

### 3.3 Extension Rate of Straight Jet Determined by Light Scattering

Owing to charge interference of the jet solution with a microscope, optical microscopy is not suitable for the measurement of the extremely small diameters of charged jets during electrospinning. To resolve this limitation, light scattering from the straight jet at different positions (*z*) from the needle tip (*z* = 0) was performed to obtain the scattering patterns (Figure 3b) of the PVA98 jet. An equatorial streak pattern associated with the straight jet appeared at z ≤ 4.0 mm, above which multiple streaks appeared around the equator (Figure 3d). The appearance of multiple streaks indicates the rapid change in orientation of the jet axis, defined as *Oz* axis in Figure 3a, with respect to the *Oz* axis within the recording time (ca. 9 ms), thus inferring the onset of jet whipping. The typical equatorial intensity profiles *I*(*q*) of the straight jet at *z* = 1.6 and 4.0 mm are shown in Figure 3c, where *q* is the magnitude of the scattering vector. Further, at *z* = 1.6 mm, *I*(*q*) exhibits three distinct intensity maxima at *q*, denoted as *q*_m1_, *q*_m2_, and *q*_m3_, from low to high *q* values, and the plateau intensity at the low-*q* region is denoted as *I*_0_ (Figure S5). Furthermore, Figures S6 and S7 show similar results for the PVA88 solution. Based on the Mie theory of scattering from a single cylinder, *q*_m1_ was used to determine *d*_j_(*z*) via a simple equation: β/*q*_m1_(*z*), where β is a constant depending on the refractive index of the polymer solution.^6^ As *z* increased, *q*_m1_ shifted to the high *q* region, indicating a decreased *d*_j_(*z*). The profiles of *d*_j_(*z*) and the cone width as functions of *z* for both electrospinning solutions (PVA98 and PVA88) are shown in Figure S8. The cone-jet transition zone was denoted as Region I, the width of which was measured from the optical image, since Mie theory of cylinder scattering was not applicable for the tapering jet with large curvatures within the irradiated domains of light scattering. Moreover, the limited resolution of the optical image might be reached to measure the jet width smaller than 50 μm for the present setup.^12^ After determining *d*_j_(*z*) at a given flow rate (*Q*), the jet velocity *v*_j_(*z*) is derived using 4*Q*/π*d*_j_,^2^ as the flowing time is extremely short (<1 ms) in the straight-jet regime, allowing for the negligibility of solvent evaporation. In this context, the extension rate, $\overset{˙}{\varepsilon }\left(z\right)$ , is calculated by d*v*_j_(*z*)/d*z*.

**Figure 3:**
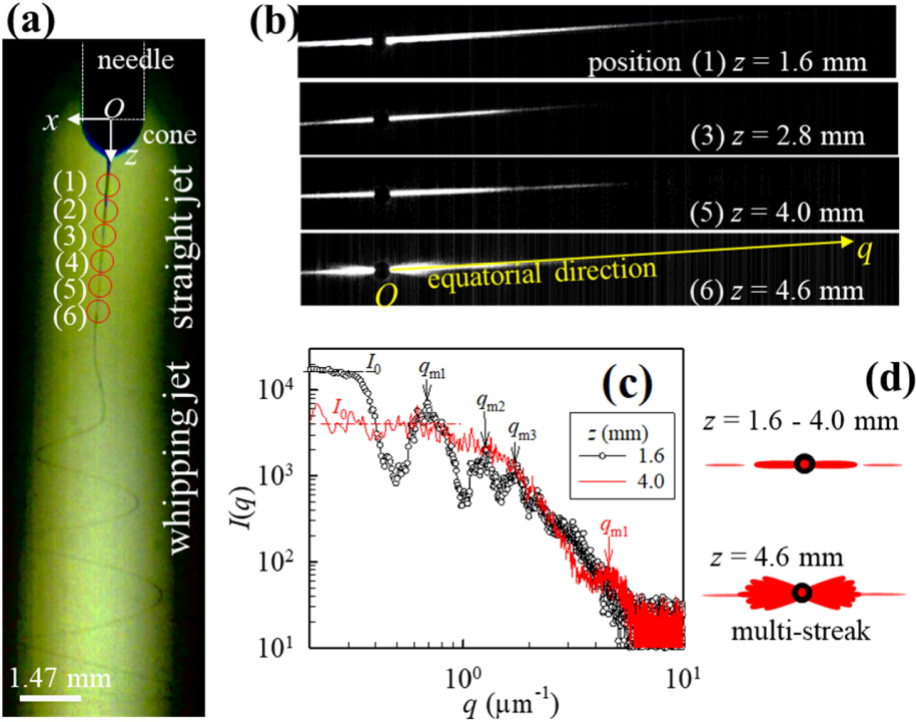
Light scattering of the straight jet. (a) High-speed videography of the electrospinning jet typically exhibits three fluid-flowing regions (i.e., the Taylor cone at the needle tip, a straight jet, and the whipping jet) during electrospinning, as the charged droplet emerging from the needle spinneret is subjected to a high electric field for electric stretching. A polarized incident laser beam (diameter = 0.4 mm; red circles) was irradiated in the straight-jet region at different positions (*z*) to obtain the light scattering patterns. The *Ox* and *Oz* axes are the coordinate fixed to the jet. (b) Scattering patterns of the PVA98 solution jet at *z* = 1.6, 2.8, 4.0, and 4.6 mm, respectively, along the *Oz* axis set along the flow direction of the jet in the spinline. (c) Equatorial intensity profiles of the jet at *z* = 1.6 and 4.0 mm. Based on the Mie scattering of a cylinder, *d*_j_(*z*) could be determined from the given intensity profiles. (d) Schematic of the scattering patterns at *z* = 1.6–4.0 and 4.6 mm. The former shows a single streak pattern with intensity maxima on the equator, and the latter exhibits multiple streaks around the beam center, owing to the presence of the whipping jet. Thus, with the increasing *z*, the first appearance of the multistreak scattering pattern could be used to precisely determine the onset of jet whipping.

Figure 4 shows the *z*-dependence of *d*_j_(*z*), *v*_j_(*z*), and $\overset{˙}{\varepsilon }\left(z\right)$ of the straight jet comprising the 7 wt % PVA88 and PVA98 solutions. Three regions (I, II, and III) had been defined to classify the characteristics of the straight jet.^6^ Regarding the PVA98 solution jet, the position of the apex of the cone from the needle tip was 1.15 mm (Figure S9), and *d*_j_ decreased with increasing *z* in Regions I and II, finally reaching a constant diameter *d*_j,e_ in Region III. In Region II, a power-law relation, $d_{j}\left(z\right)\propto z^{-n}$ , was observed, exponent *n* was derived as 1.82, and *d*_j,e_ in Region III was determined as ca. 2 μm. The derived *n* significantly exceeded that for the PNIPAM/DMF solution,^6^ i.e., 0.5, indicating more dramatic jet stretching for the more conductive PVA aqueous solution. Notably, *v*_j_(*z*) reached a large value (15 m/s) after the fluid element traveled a short distance (5 mm). The corresponding $\overset{˙}{\varepsilon }\left(z\right)$ increases from 10 ^3^ to 10^4^ s^–1^ within a very short time (0.67 ms; Figure 4c). These $\overset{˙}{\varepsilon }\left(z\right)$ values exceeded the rheometer-determined intrinsic relaxation rate of polymer chains ( $\tau_{d}^{-1}$ , ∼833 s^–1^; Table 1). Regarding the PVA88 solution jet, Region III was not detected, the derived *n* was 2.12, and *v*_j_ reached 15 m/s after traveling over a distance of 4 mm, so that the averaged $\overset{˙}{\varepsilon }\left(z\right)$ exceeded that of the PVA98 jet. To quantitatively characterize the effectiveness of chain orientation (or stretching) in a flow field, the Weissenberg number (Wi = $\overset{˙}{\varepsilon }\tau_{d}$ ) was used.^17^ Given that Wi exceeded 1.0 (i.e., $\overset{˙}{\varepsilon }$ > $\tau_{d}^{-1}$ ), the nonlinear viscoelastic behavior of the solution jet must be applied, and the chain orientation would play a crucial role during electrospinning. At *z =* 3 mm, the derived Wi numbers were 6 and 10 for the PVA98 and PVA88 jets, respectively. The high Wi number supports the findings in Figure 2 that flow-induced phase separation of the one-phase solution does occur in the straight jet before jet whipping.

**Figure 4:**
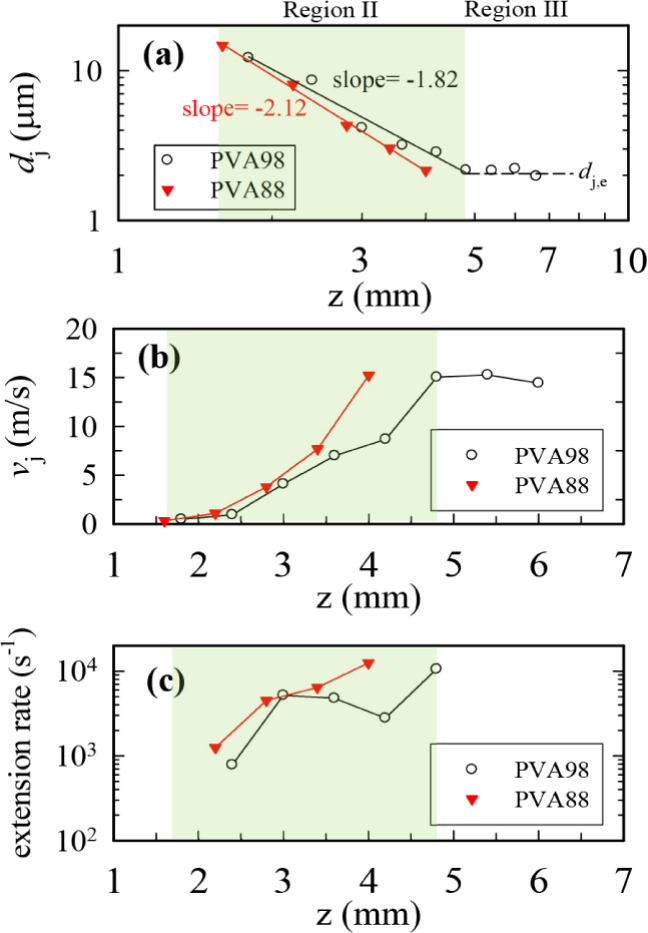
(a) Variation in the straight-jet diameter (*d*_j_) as a function *z* along the straight jet during the electrospinning of the 7 wt % PVA solutions, (b) *z*-dependence of *v*_j_, and (c) *z*-dependence of $\overset{˙}{\varepsilon }\left(z\right)$ in the straight jet. Region II is highlighted in a green shade. The jet obtained from the PVA98 solution exhibited a constant *d*_j_(*z*) value at the straight-jet end (Region III), with a constant *v*_j_(*z*) of ∼15 m/s so that $\overset{˙}{\varepsilon }\left(z\right)$ is approximately zero in this region before the jet whipping process. Conversely, Region III for the straight jet of the PVA88 solution could not be detected.

### 3.4 Flow Field Inside the Taylor Cone Observed by Particle Image Velocimetry

The schematic setup for particle image velocimetry is shown in Figure S3. The experiment was performed to trace the particle image (Figure S10), and the details are shown in Movie S1. The flow field inside the Taylor cone exhibits transient behavior due to the external perturbations at the cone/air interface. Backflow was observed in the central part of the Taylor cone, and two vortex groups coexisted in the Taylor cone along the focusing plane (Figure 5a). One vortex was right-handed (RH; with a clockwise rotation), and the other vortex was left-handed (LH; with counterclockwise rotation), similar to the toroidal vortex observed during electrospraying.^13–15^ By tracing the time dependence of particle position (Figure S10), the backflow velocity was determined to be $3\times 10^{-4}$ m/s, which was ca. 5-fold higher than the fluid velocity in the needle spinneret (= 4*Q*/ $\pi D_{i}^{2}$ , *D*_i_ is the inner diameter of the needle). However, the fluid velocity is much lower than the straight-jet velocity, and the derived extension rate is extremely small, so that the flow-induced phase separation seems unlikely to occur in the Taylor cone. The dynamic balance of these opposite vortices visually stabilizes the Taylor cone (Figure S8). Thus, fluid flows into the apex region (the bottom part of the toroidal vortices) from the vicinity of the cone surface are subjected to the highest electric field-induced shear stresses.^14^ In the bottom part of the main vortices, the fluid elements might exhibit radial velocity (*v*_r_) and circumferential velocity (*v*_θ_) toward the cone apex, as shown in Figure 6b. Further, the *v*_θ_ component might generate a “swirling flow” into the cone apex. Thus, the fluid elements with *v*_θ_ > 0 and *v*_θ_ < 0 would produce LH and RH swirls, respectively (Figure 6). Notably, no swirl was observed at *v*_θ_= 0. Notably, intensive stretching and swirling occur as the fluid elements ( $v_{\theta }\neq 0$ ) pass the narrow channel of the cone apex exhibiting the highest electric field (ca. 10^3^ kV/m), calculated numerically by the finite element method to analyze the electric field in the electrospinning space.^18^ The swirl may produce a helical vortex when propagating along the axial direction of the straight jet. Observing the dynamic interchange of the RH and LH swirls entering the cone apex is crucial. In other words, the intermittent RH (or LH) swirl flows into the apex to be subsequently brought into the straight-jet region. After being subjected to a high electric field at the cone-jet transition zone (Region I), the highly twisted jet can be feasibly solidified in a nonsolvent reservoir containing 1-propanol for observation (Figure S11). The knotty state of the strings, as observed in Figure S11, implies that the strings are twisted to a sufficiently large degree after they are formed in the straight jet via flow-induced phase separation.

**Figure 5:**
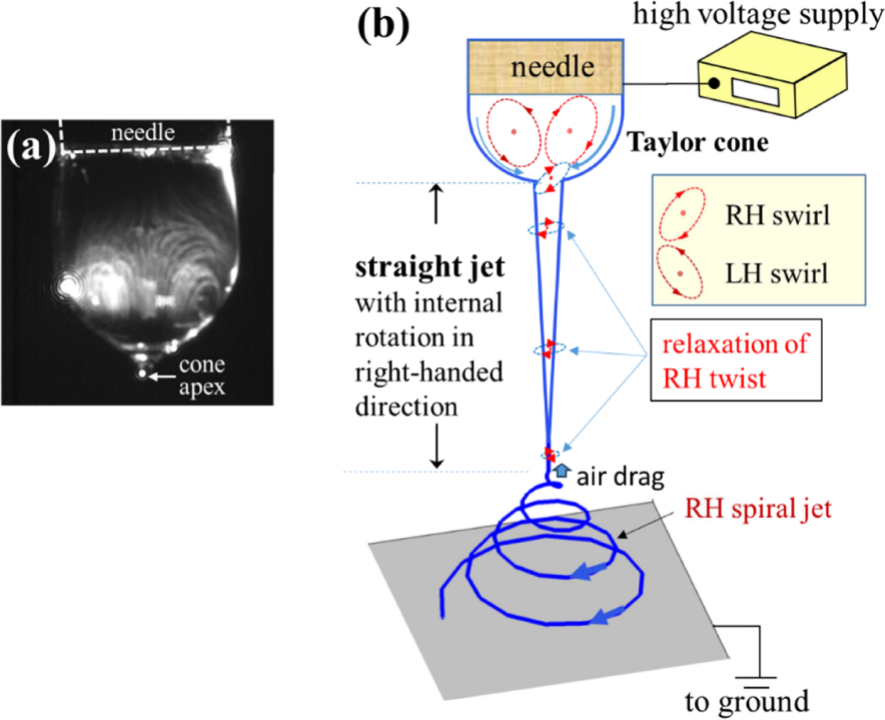
Fluid flow in the Taylor cone and spiral jet. (a) Vortex flow in the Taylor cone determined by particle image velocimetry; the details are shown in Movie S1. (b) Schematics of the electrospinning jet to illustrate the Taylor cone, straight jet, and spiral jet. In the cone, opposite vortices coexist to dynamically alter the flow field and facilitate backflow in the central part of the cone, whereas the fluid elements enter the apex region via the region between the vortex and bottom-cone surface, as shown by the blue curved arrows. As illustrated, an RH swirl enters the cone apex, and the residual vorticity at the straight-jet end generates the RH spiral jet. It is noted that the air drag at the straight jet end is high to induce a buckle^6^ to initiate the jet spiraling.

**Figure 6:**
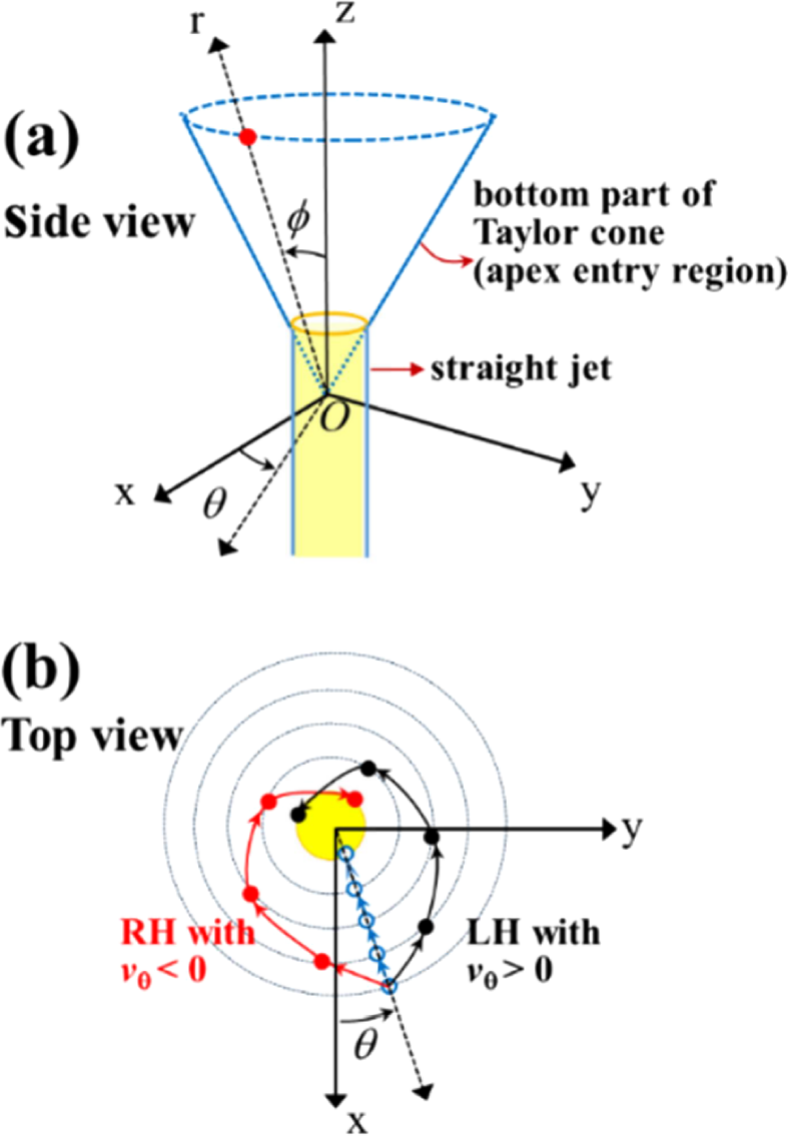
(a) Spherical coordinates (*r*, θ, ϕ) for the entry region from the Taylor cone to straight jet, i.e., the cone apex. The yellow cylinder represents the straight jet. (b) Motion of fluid elements with or without circumferential velocity (*v*_θ_). The red arrows show the streamline of fluid elements with *v*_θ_ < 0, yielding the right-handed rotation flow (RH vortex or swirl) toward the cone apex, whereas the black arrows show the streamline of fluid elements with *v*_θ_ > 0, yielding the left-handed rotation flow (LH swirl or vortex). The blue arrows show the streamline of the fluid element with radial velocity (*v*_r_) and *v*_θ_ = 0.

### 3.5 High-Speed Videography to Obtain Rotation Direction and Extension Rate of the Spiral Jet

At the straight-jet end, the charged jet started to deviate from the straight path. This deviation was caused by the compressive force of air drag^6,7,19^ to induce a buckle, followed by the Coulomb repulsion^2,7^ between the buckled-jet segments. This is likely followed by the in-plane or out-of-plane vibrations of the charged jet (Figure S12). The out-of-plane jet vibration might produce a spiral jet (Movie S2), which rotates clockwise (RH) or counterclockwise (LH) depending on the velocity field of the entry flow at the cone apex.

As illustrated in Figure 5b, when an RH vortex flows into the cone-jet transition zone where the electric field is highly concentrated,^18^ a more enhanced RH vortex is produced. Subsequently, the fluid elements in the main straight jet should rotate clockwise (RH) to release the imposed torsion, as no rotational electric field is applied to maintain the RH vorticity. At the straight-jet end, the subsequent bending jet will exhibit the RH spiral motion if the preimposed torsion is not fully released. Similarly, as an LH vortex enters the cone apex, the straight jet might rotate and evolve, exhibiting the LH spiral jet to release the residual LH twist. Remarkably, a transition of the handedness (RH ⇌ LH) of the spiral jet is intermittently observed by high-speed video at a frame rate of 8000 fps (Movie S2). A similar RH ⇌ LH transition of the spiral motion has been reported by Reneker’s group using stereography combined with appropriate jet illumination.^20^ The dried fibers might exhibit a helical configuration on the grounded collector if the residual torsion is not fully released after solvent evaporation. Helical fibers with inverted handedness, posing opposite twisting directions, have been observed.^21^

Figure 7 shows snapshots of the spiral jet of the PVA88 solution at a frame rate of 8000 fps. The axial velocity (*v*_z_) of the spiral jet can be derived by measuring the time dependence of the axial displacement of the first wave of the bend with respect to the straight-jet path. Similarly, the lateral velocity (*v*_r_) of the spiral jet is determined from the time dependence of the lateral displacement. These dynamic analyses are illustrated in Figures S13 and S14, and the results are shown in Table 2. In this study, the values of *v*_z_ and *v*_r_ are approximately 4.5–4.8 and 2.4–2.9 m/s, respectively. Based on ref 7, the spiral frequency *f* is obtained from the ratio of *v*_z_/λ. The maximum extension rate for spiral motion is calculated by ${\overset{˙}{\varepsilon }}_{\max}=\frac{\pi v_{r}v_{z}}{\lambda \sqrt{v_{r}^{2}+v_{z}^{2}}}$ . These values yield a maximum extension rate of 2700 s^–1^ for the spiral jet of the PVA88 solution (Table 2), exceeding that of the PVA98 solution (1600 s^–1^). Thus, the extension rate of the spiral jet is still higher than the relaxation rate of the solution. Occasionally, the spiral jet is stretched to break at the weak points in the phase-separated string structures, and this phenomenon is illustrated in Figure S15.

**Figure 7:**
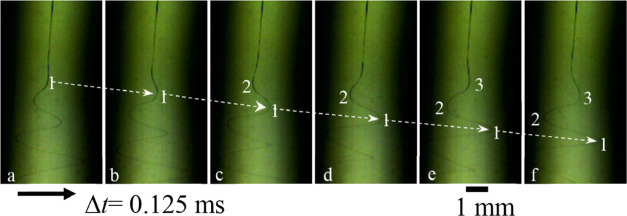
Six consecutive snapshots of the PVA88 solution jet undergoing spiral motion. Numbers 1, 2, and 3 indicate the position of the bend positions of the first, second, and third wave generations, respectively. The initial velocity of the spiral jet, the bending frequency, and the maximum $\overset{˙}{\varepsilon }\left(z\right)$ of the spiral jet can be determined from the detailed analyses of these images (Figures S13 and S14).

**Table 2:** Cone Height *H*_c_, Jet Length *L*_j_, and Characteristics of the Bending Jet (Wavelength λ, Axial Velocity *v*_*z*_, Lateral Velocity *v*_r_, Bending Frequency *f*, and Maximum Extension Rate ${\overset{˙}{\varepsilon }}_{\max}$ )

solution code	*H*_c_ (mm)	*L*_j_ (mm)	λ/2 (mm)	*v*_*z*_ (m/s)	*v*_r_ (m/s)	*f* (Hz)	${\overset{˙}{\varepsilon }}_{\max}$ (s^–1^)
PVA88	0.98	5.48 ± 0.57	1.25 ± 0.37	4.55	2.39	1820	∼2700
PVA98	1.15	8.53 ± 1.04	2.54 ± 0.68	4.81	2.94	950	∼1600

Helical (or twisted) fibers are not uncommon;^21^ they are sometimes observed coexisting with straight fibers. This is demonstrated in Figure 8a for the as-spun PVA fibers, which exhibit a large bundle of twisted nanofibers with a diameter of ca. 1 μm, together with straight nanofibers with a diameter of 200–800 nm. Helical fibers are also observed for the polyethylene fibers (Figure 8c) and Nylon-6 fibers (Figure 8d), which are associated with the twisted jet, resulting from the upstream swirling flow at the cone apex. The processing conditions used to obtain these fibers were (1) polyethylene fibers:^22^ 8 wt % in o-DCB solvent, electrospun at 100 °C under the processing variables of *Q* = 1.0 mL/h, *V* = 17 kV, and *H* = 14 cm; and (2) Nylon-6 fibers:^23^ 7 wt % in formic acid solvent, electrospun at room temperature with *Q* = 0.3 mL/h, *V* = 20 kV, and *H* = 7 cm.

**Figure 8:**
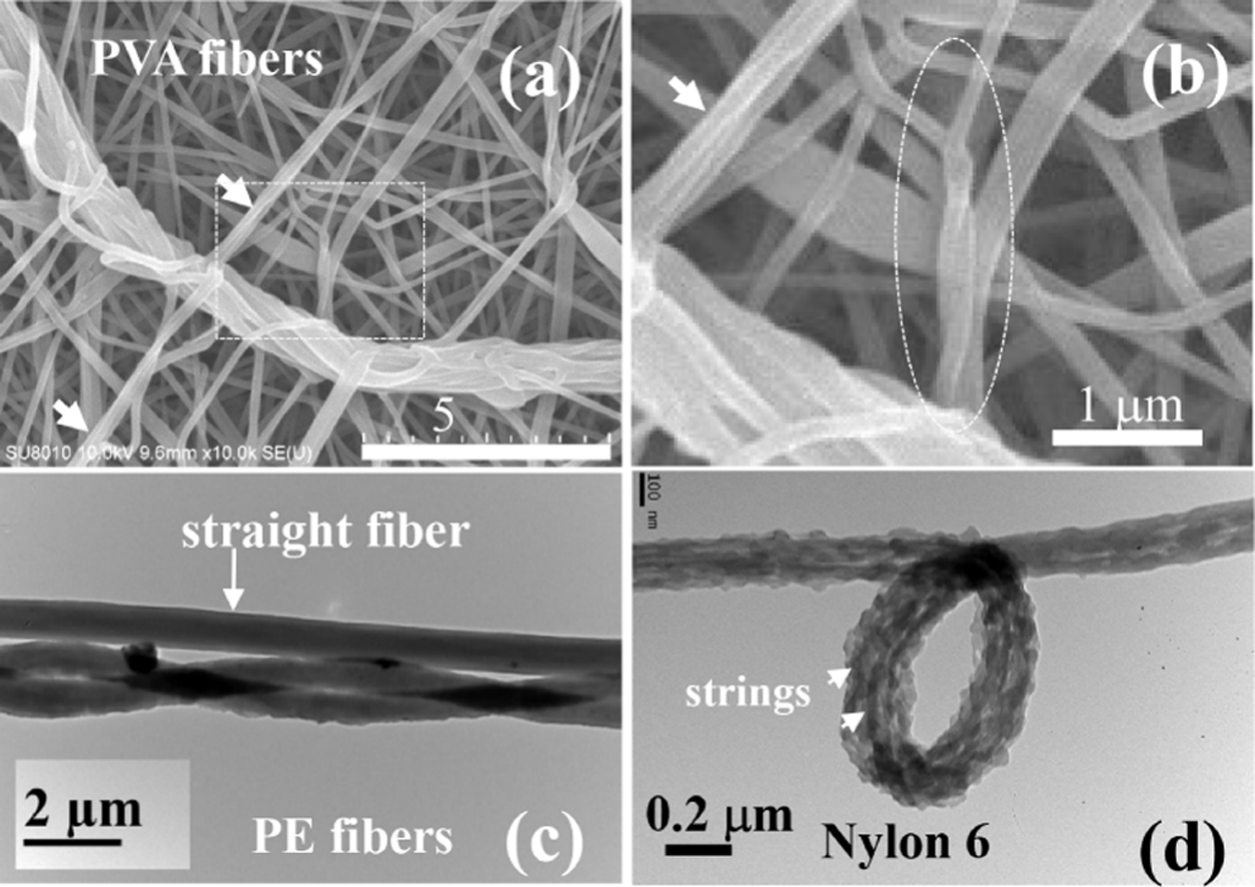
Twisted nanofibers observed on the grounded collector. (a) SEM image of PVA98 fibers with twisted fiber bundles; the dashed box is enlarged to show in (b). In parts (a) and (b), the arrows show the lateral association (i.e., fasciation) of small fibers. In (b), two intertwined small fibers are shown in the oval. (c) TEM image of polyethylene fibers exhibiting one straight fiber with two twisted fibers. (d) TEM image of a knotted Nylon-6 fiber composed of three strings.

### 3.6 Origin of the Spiral Jet

The downstream spiral motion of the bending jet is essential to the twisted jet, which attempts to relax its imposed torsion, as there is no “rotation electric field” in the straight-jet region to maintain the exerted vorticity. Thus, inherent relations exist among the flow field in the Taylor cone (Figure 5a and Movie S1), the twisted jet (Figure 2d), the spiral direction of the bending jet (Movie S2), and the helical dried fibers on the grounded collector (Figure 8a). It is intriguing to discover that the downstream spiral jet is relevant to the upstream entry flow at the cone apex; a subject that resembles the classic extrusion instability,^24–26^ i.e., the periodic swirling flow developed in a contraction upstream might induce the helical distortion of the extrudates at the expansion downstream.

### 3.7 Hydrolysis Effects of PVA on Jet Stability and Fiber Morphology

As the hydrolysis level of PVA increases from 88% to 98%, the electric conductivity and surface tension of PVA/water solutions increase but the zero-shear viscosity and relaxation time decrease. For these two electrospinning solutions subjected to the same processing parameters of *Q, V*, and *H*, the PVA98 jet possesses a larger cone height and longer straight-jet length than the PVA88 jet, whereas the straight-jet-end diameter (*d*_j,e_) is similar at 2 μm. The extension rate of the jet in the straight-jet section is higher than the relaxation rate of the solution, and hence, the phase-separated structures of the strings have been validated in both PVA88 and PVA98 jets.

To control the final fiber morphology, stretching stresses of the jet also play an important role. In principle, the stretching stresses of a straight jet with a diameter of *d*_j_(*z*) along the spinline at the coordinate *z,* starting at the needle tip, can be estimated by σ(z)E(z) , where σ(z) is the surface charge density and E(z) is the electric field of the jet at the given *V* and *H* setup by the needle-plate electrodes. After acquiring the electric current *I*, the σ(z) of the given jet section can be deduced by $\frac{Id_{j}\left(z\right)}{4Q}$ , which in turn gives rise to the stretching stresses expressed by $\frac{Id_{j}\left(z\right)E\left(z\right)}{4Q}$ . For the needle-plate configuration, our numerical calculation results^18,27^ show that *E*(*z*) is a position function and is highly concentrated at the needle tip and exponentially decays along the *z*-direction; in other words, the jet segment far from the needle tip is subjected to a low *E* for electrical stretching.

At the straight jet end where *z* = *L*_j_, the electric stress is reduced to be $\frac{Id_{\mathrm{j.e}}E\left(z=L_{j}\right)}{4Q}$ . Thus, the stretching stresses at the straight jet end depend on the measured quantities of *I*, *d*_j,e_, *Q*, and *E* (*z* = *L*_j_). Moreover, the measured current has been found to scale with processing parameters.^28^ Thus, the stretching stresses cannot be independently controlled since their magnitude is relevant to the processing parameters (*Q*, *V*, and *H*) as well as the solution properties (γ, κ, and η).

For the present two solutions subjected to the same processing parameters of *Q*, *V*, and *H*, the PVA88 jet possesses a shorter *L*_j_ (so that it is subjected to a higher *E* for stretching) and a larger *I* than the PVA98 jet. Moreover, both jets have a similar *d*_j,e_ at the straight jet end. These experimental results indicate that the electrical stresses to stretch the PVA88 jet are higher than those for the PVA98 jet. However, the average diameter of the PVA88 fibers is larger on the grounded collector. One may attribute this unexpected result to the large zero-shear viscosity of the PVA88 solution, which resists electric stretching. This deduction is based on the incorrect assumption that the one-phase solution jet is continuously stretched before fiber formation on the grounded collector. As demonstrated previously, flow-induced phase separation inevitably occurs in the PVA jet to produce phase-separated structures of the strings with various diameters. The lateral association (i.e., fasciation^12^) of these strings may play a critical role in controlling the final fiber diameter. The fasciation is mainly induced by the Poissonian contraction of the highly stretched jet, forcing the neighboring liquid strings closer to one another to develop the overlapped region. This leads to our proposition that, due to the high hydrolysis of PVA98, the interchain association in the PVA98 jet is enhanced to readily facilitate the string fasciation, thereby reducing the final fiber diameter after solvent removal.

## 4 Conclusion

Here, we directly investigated jet behaviors to validate the flow-induced phase separation in a straight jet on the basis of two experimental findings: (1) the straight jet is not homogeneous with respect to concentration but is composed of assembled strings with various widths in the matrix solution as a consequence of the flow-induced phase separation; this is revealed by examining the frozen jet in liquid nitrogen after freeze-drying, and (2) the extension rate of the solution in the straight jet is higher than the relaxation rate of the polymer solution; the derived Wi number is sufficiently high to trigger the flow-induced phase separation. In addition, the dynamic vortex flow in the Taylor cone may intermittently introduce a swirl with different handedness to the cone-jet transition zone and subsequently to the straight jet, causing the straight jet to twist to release the imposed torsion. The rotating swirl in the straight jet controls the spiral motion of the bending jet downstream as the flowing jet deviates from its linear path induced by the air drag.^6,19^ In other words, the rotational direction of the spiral jet depends upon the handedness of the upstream swirl flowing into the cone apex. Remarkably, a transition of the handedness of the spiral jet is intermittently observed by high-speed videography, which is predicted to reflect the intermittent entry of swirls with different handedness into the cone apex.

Our results show that the existing knowledge, assuming the one-phase-jet flow in the spinline until jet drying due to solvent evaporation, is not appropriate to account for the fiber formation during electrospinning. In contrast, flow-induced concentration fluctuations that further trigger and proceed with solution phase separation^12^ must be considered to reveal the extremely dynamic and nonequilibrium process of electrospinning.

## Supplementary Material







## Notes

Schematics for the collection of straight jet during electrospinning (Figure S1); schematic sketch of the light scattering setup (Figure S2); schematic setup for particle image velocimetry measurement (Figure S3); SEM images and fiber diameter distribution of as-spun PVA fibers (Figure S4); light scattering results of the PVA98 solution (Figure S5); light scattering profiles of the PVA88 solution at different *z* (Figure S6); light scattering results of the PVA88 solution (Figure S7); profiles of the Taylor-cone width and jet diameter as a function of *z* for the PVA98 and PVA88 solutions (Figure S8); images of Taylor cone and straight jet to determine *H*_c_ and *L*_j_ (Figure S9); particle image velocimetry of PVA88/H_2_O in the Taylor cone (Figure S10); SEM images of highly twisted jet collected in nonsolvent of 1-propanol (Figure S11); illustrations of in-plane and out-of-plane lateral vibration of charged jet (Figure S12); fluctuations of *L*_j_ and λ/2 of the straight-jet end (Figure S13); determination of the axial and lateral velocity of the spiral jet (Figure S14); and consecutives snapshots of high-speed videography to show the breaking of spiral jet (Figure S15) (PDF)
Particle image velocimetry in the Taylor cone (MP4)
High-speed videography of the spiral jet (MP4)

## References

[1] Chae H. G., Kumar S. (2008). Making strong
fibers. Science.

[2] Reneker D. H., Yarin A. L. (2008). Electrospinning
jets and polymer nanofibers. Polymer.

[3] Hohman M. M., Shin M., Rutledge G., Brenner M. P. (2001). Electrospinning
and electrically forced jets. II. Applications. Phys. Fluids.

[4] Agarwal S., Greiner A., Wendorff J. H. (2013). Functional
materials by electrospinning
of polymers. Prog. Polym. Sci..

[5] Xue J., Wu T., Dai Y., Xia Y. (2019). Electrospinning and electrospun nanofibers:
Methods, materials, and applications. Chem.
Rev..

[6] Wang Y., Hashimoto T., Li C. C., Li Y. C., Wang C. (2018). Extension
rate of the straight jet in electrospinning of poly­(N-isopropylacrylamide)
solutions in dimethylformamide: Influences of flow rate and applied
voltage. J. Polym. Sci., Part B: polym. Phys..

[7] Wang C., Hashimoto T., Wang Y. (2021). Extension rate and bending instability
of electrospinning jets: Tthe role of the electric field. Macromolecules.

[8] McKee M. G., Wilkes G. L., Colby R. H., Long T. E. (2004). Correlations
Of
Solution Rheology With Electrospun Fiber Formation Of Linear And Branched
Polyesters. Macromolecules.

[9] Malkin A. Y., Arinstein A., Kulichikhin V. G. (2014). Polymer extension flows and instabilities. Prog. Polym. Sci..

[10] Wingstrand S. L., Imperiali L., Stepanyan R., Hassager O. (2018). Extension induced phase
separation and crystallization in semidilute solutions of ultrahigh
molecular weight polyethylene. Polymer.

[11] Wang C., Hashimoto T. (2018). Self-organization
in electrospun polymer solutions:
Ffrom dissipative structures to ordered fiber structures through fluctuations. Macromolecules.

[12] Wang C., Hashimoto T. (2020). A scenario of a fiber formation mechanism
in electrospinning:
Jet evolves assemblies of phase-separated strings that eventually
split into as-spun fibers observed on the grounded collector. Macromolecules.

[13] Hayati I., Bailey A. I., Tadros T. F. (1986). Mechanism of stable
jet formation
in electrohydrodynamic atomization. Nature.

[14] Barrero A., Ganán-Calvo A. M., Dávila J., Palacio A., Gomez-González E. (1998). Low and high
Reynolds
number flows inside Taylor cones. Phys. Rev.
E.

[15] Herrada M., López-Herrera J. M., Gañán-Calvo A. M., Vega E. J., Montanero J. M., Popinet S. (2012). Numerical simulation
of electrospray in the cone-jet mode. Phys.
Rev. E.

[16] Bellan L. M., Craighead H. G., Hinestroza J. P. (2007). Direct measurement of fluid velocity
in an electrospinning jet using particle image velocimetry. J. Appl. Phys..

[17] Larson R.
G., Desai P. S. (2015). Modeling
the rheology of polymer melts and solutions. Annu. Rev. Fluid. Mech..

[18] Wang C., Chien H.-S., Hsu C.-H., Wang Y.-C., Wang C.-T., Lu H.-A. (2007). Electrospinning
of polyacrylonitrile solutions at elevated temperatures. Macromolecules.

[19] Taylor G. (1969). Electrically
driven jets. Proc. R. Soc. Lond..

[20] Liu K., Ertley C. D., Reneker D. H. (2012). Interpretation
and use of glints
from an electrospinning jet of polymer solutions. Polymer.

[21] Silva P. E. S., de Abreu F. V., Godinho M. H. (2017). Shaping helical electrospun filaments:
A review. Soft Matter.

[22] Chen, C.-C. Electrospun polyethylene and its composite nanofibers and its property characterization. Master thesis, National Cheng Kung University. Taiwan 2012.

[23] Tsou S.-Y., Lin H.-S., Wang C. (2011). Studies on the electrospun Nylon
6 nanofibers from polyelectrolyte solutions: 1. Effects of solution
concentration and temperature. Polymer.

[24] Boger, D. V. ; Walters, K. Rheological phenomena in focus; Elsevier: Amsterdam, 1993.

[25] Nigen S., Kissi N. E., Piau J.-M., Sadun S. (2003). Velocity field for
polymer melts extrusion using particle image velocimetry: Stable and
unstable flow regimes. J. non-Newtonian Fluid
Mech..

[26] Denn M. M. (2004). Fifty years
of non-Newtonian fluid dynamics. AIChE J..

[27] Wang C., Cheng Y. W., Hsu C. H., Chien H. S., Tsou S. Y. (2011). How to
manipulate the electrospinning jet with controlled properties to obtain
uniform fibers with the smallest diameter?—a brief discussion
of solution electrospinning process. J. Polym.
Res..

[28] Theron S. A., Zussman E., Yarin A. L. (2004). Experimental investigation of the
governing parameters in the electrospinning of polymer solutions. Polymer.

