# Supplementary material for: Novel Insights into Solution Electrospinning for Nanofibers
Source: Macromolecules. 2025 Jun 17;58(13):6762–70. doi: 10.1021/acs.macromol.5c00703 (PMC12257595; doi:10.1021/acs.macromol.5c00703)
Supplement: Supplementary file 1 [file ma5c00703_si_001.pdf]

Supporting Information for

Novel Insights into Solution Electrospinning for Nanofibers

Chi Wang[*a], Pin-Hsien Lu[a], Yin-Chuan Kuo[a], Chih-Hsien Kuo[a], Hsin-Yi Lai[a],*

Shao-Hua Wu[a], Takeji Hashimoto[b]

Corresponding authors: chiwang@mail.ncku.edu.tw

The PDF file includes:

Figures. S1 to S15

Other Supplementary Material for this manuscript includes the following:

Movies S1 to S2

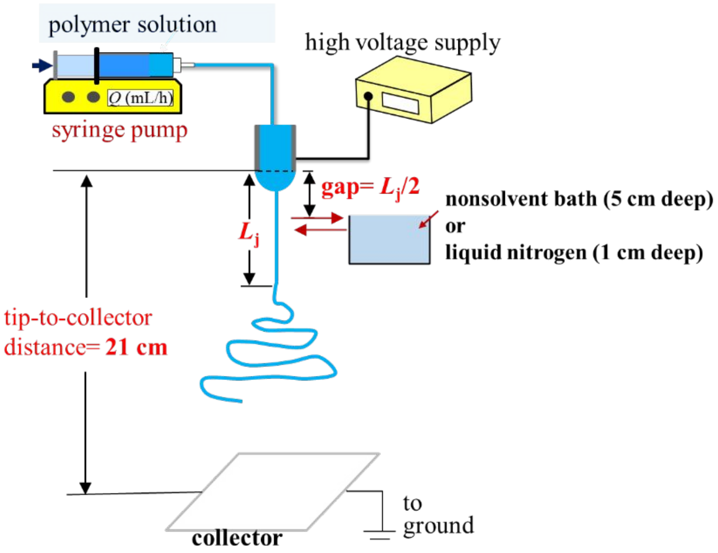

Figure S1. Schematics for the collection of straight jet during electrospinning. A moving bath containing liquid nitrogen is rapidly transferred in and out of the spin-line to collect the "straight jet" to rapidly freeze the solution jet. The sampling position is about half of the straight jet length L_j, measured from the needle tip. The bath with the collected jet and liquid nitrogen is then placed in a high vacuum oven to remove the water solvent. The residual structures are observed under SEM to provide evidence of flow-induced phase separation during electrospinning in the jet. Moreover, nonsolvent of 1-propanol is also applied to freeze the internal structures of the flowing jet.

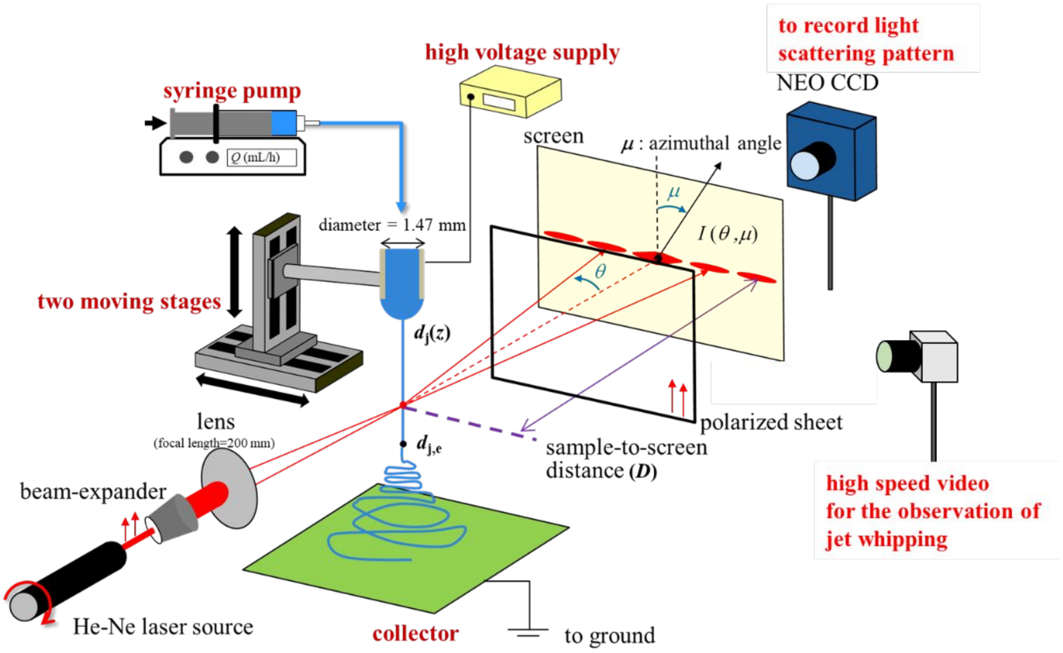

Figure S2. Schematic sketch of the light scattering setup to obtain the V_V scattering patterns of the electrospinning jet at different z positions from the needle tip (z = 0) recorded by the NEO CCD, from which the equatorial intensity profile $I(q)$ at azimuthal angle μ of 90° is extracted for further analyses. Double arrows show the polarization directions of laser beam and the polarizer sheet. The laser beam is vertically polarized along the jet direction. The electrospinning jet is controlled by two moving stages so that laser beam precisely irradiates at the desired position of the jet for capturing the corresponding scattering pattern on the screen. A high-speed video is used to observe the whipping jet.

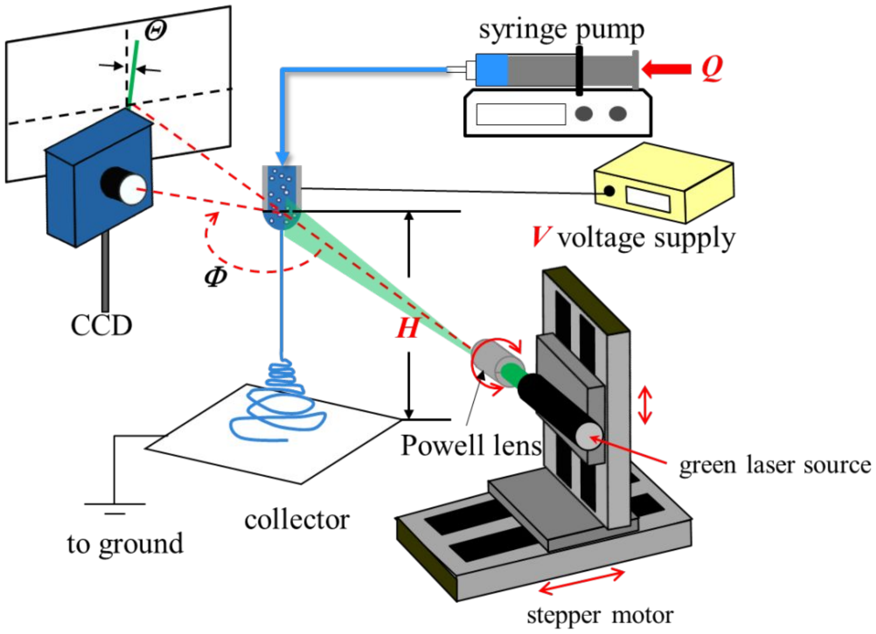

Figure S3. Schematics for particle image velocimetry to trace the fluid flow in the Taylor cone. The fan angle of the Powell lens is 5°. The 300 mW laser source has a wave-length is 0.532 μm. The observation angles of the particle flow are Θ= 45° and Φ= 135°. The observation angle Φ is defined in the horizontal plane, and the observation angle Θ is changed by rotating the Powell lens around its axis.

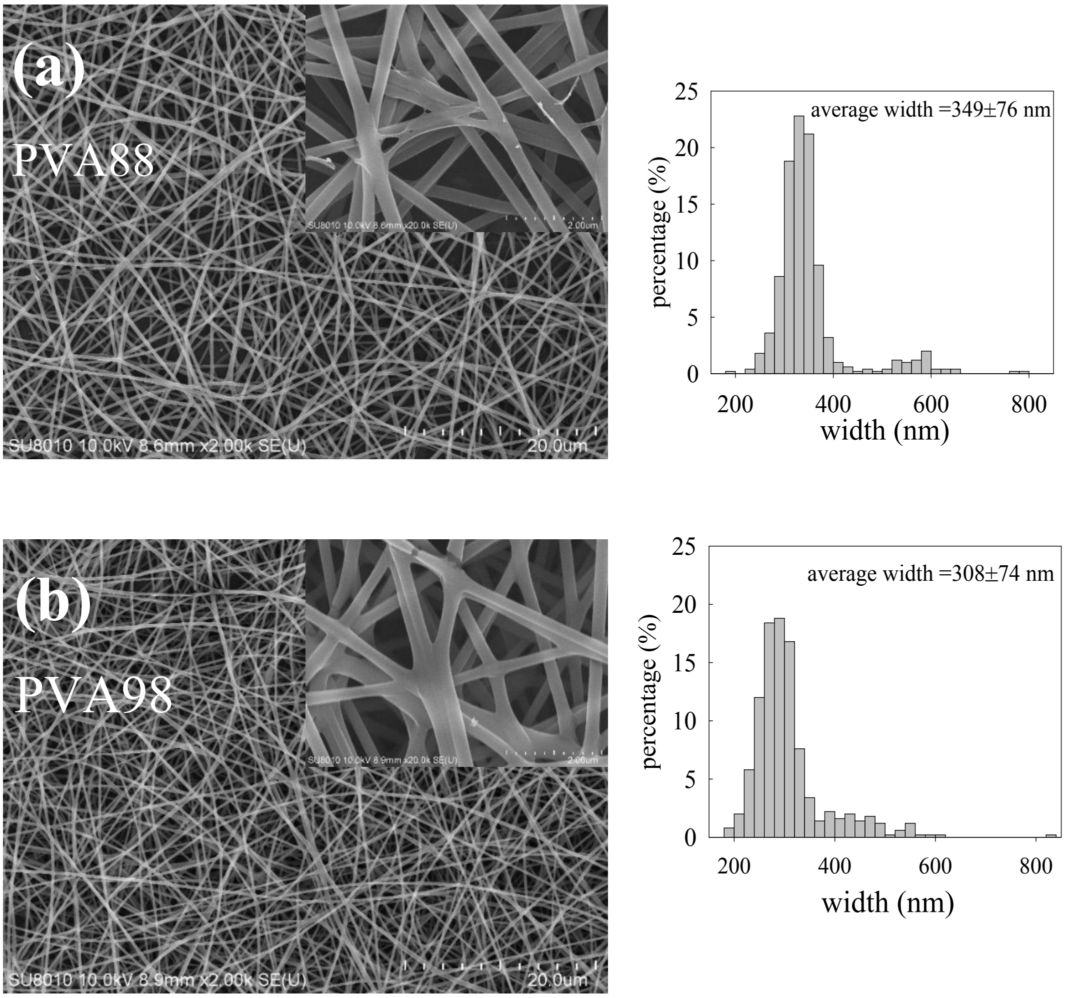

Figure S4. SEM images of as-spun PVA fibers on the grounded collector, placed at 21 cm from the needle spinneret. The histogram of fiber diameter distribution is shown in the right column, measured from the collection of 500 fibers under the magnification of 20k (shown in the insets). The average fiber diameters (d_f) are 349 $\pm$ 76 nm and 308 $\pm$ 74 nm, respectively, for the PVA88 fibers and PVA98 fibers. It seems that d_f is marginally larger for the fibers obtained from the PVA88 than from PVA98 solution in consideration of the large standard deviation with respect to the averaged value. The wide range of fiber diameter distribution is commonly observed due to plausibly different levels of string fasciation in the individual fibers before solvent removal. It is also noted that ribbon-like fibers are produced from both the PVA88 and PVA98 solutions (as typically seen in the inset in (a)); in this case, the width of the ribbon is considered.

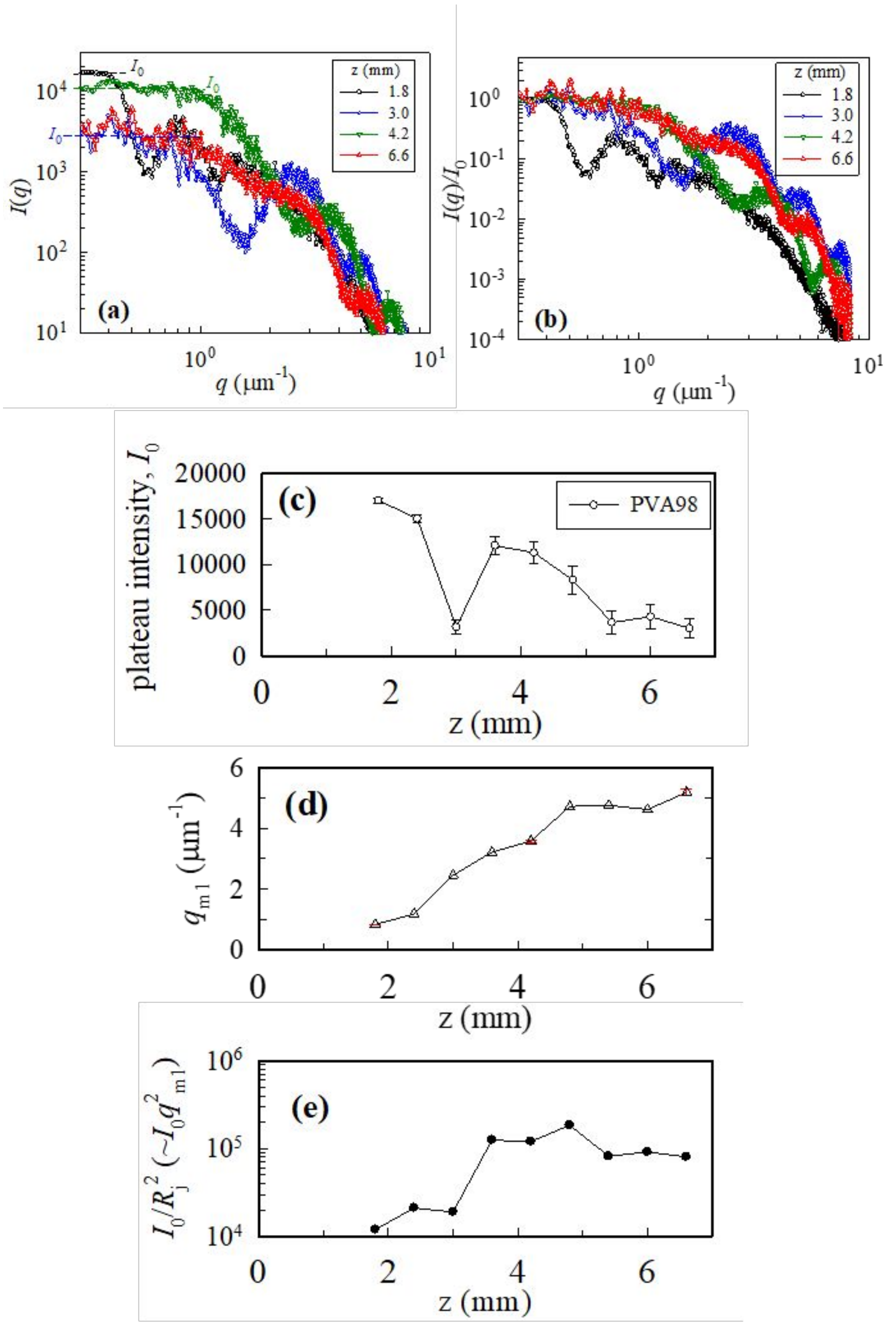

Figure S5. (a) Scattering intensity profiles of the straight jet of the 7 wt% PVA98 solution at different positions z; I_0 is the plateau intensity at low q region. (b) Plots of reduced intensity profile $I(q)/I_0$ at different jet positions. (c) Plateau intensity I_0. (d) The position value of the first scattering maximum q_{m1}. (e) the magnitude of I_0/R_j^2.

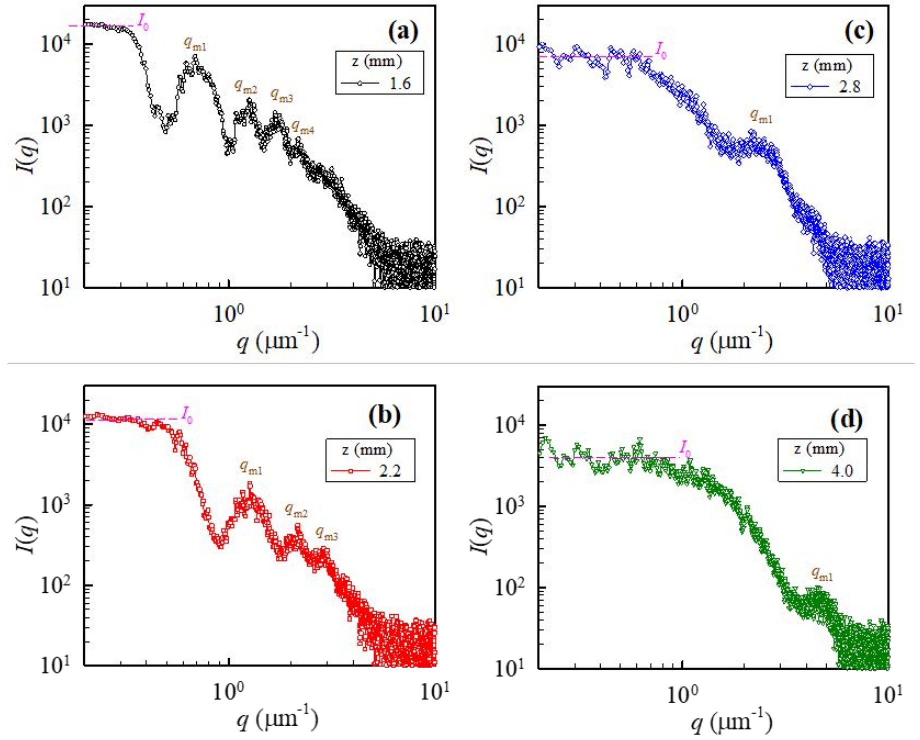

Figure S6. Equatorial intensity profile $I(q)$ versus the magnitude of scattering vector q of the straight jet at different z during electrospinning of the PVA88 solution. (a) z = 1.6 mm, (b) z = 2.2 mm, (c) z = 2.8 mm, and (d) z = 4.0 mm. Each scattering profile is corrected for instrument-induced background scattering. The plateau intensity at low q region is denoted as I_0. The peak positions for the first, second, third, and forth scattered intensity maximum are denoted as q_{m1}, q_{m2}, q_{m3}, and q_{m4}, respectively, and q_{m1} is used to calculate the jet diameter d_j by the simple relation: $d_j = \beta/q_{m1}$, where β is constant depending upon the refractive index of the electrospinning jet.

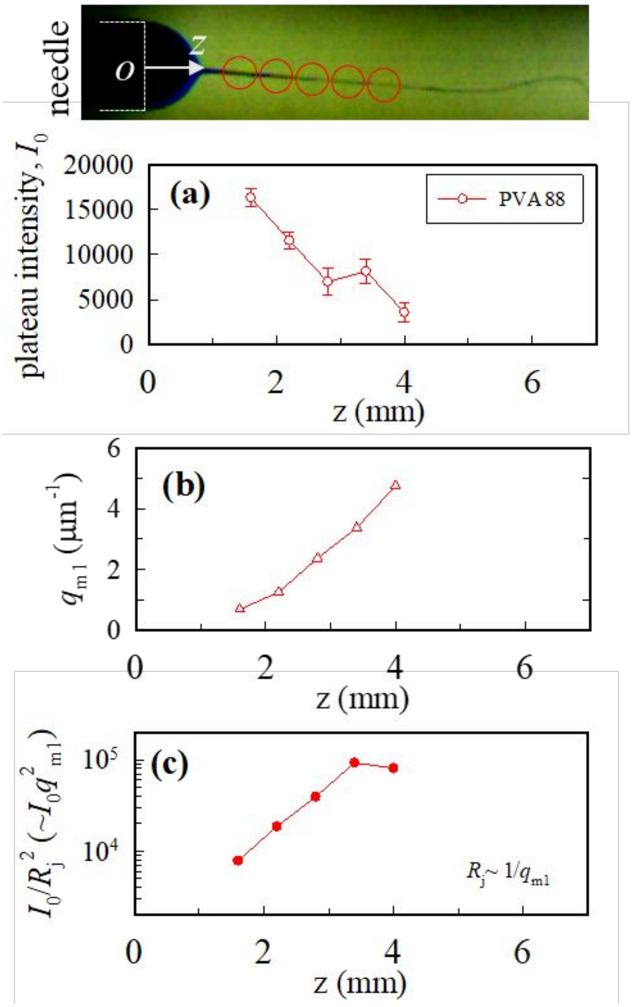

Figure S7. Light scattering results of the 7 wt% PVA88 solution to show the *z*-dependence of (a) plateau intensity I_0, (b) the position value of the first scattering maximum q_{m1}, and (c) the magnitude of I_0/R_j^2.

Based on the Mie theory for cylinder scattering[6], I_0/R_j^2 (or $I_0 q_{m1}^2$) would be constant for a homogeneous jet in the absence of internal structure. In (c), we found that the magnitude of I_0/R_j^2 is not constant, but increases by a factor of 10 in a short distance of 2 mm. It implies that the straight jet is not homogeneous but contains internal structures, the scattering of which contributes significantly to the measured I_0. In other words, the measured $I(q)$ should be composed of two contributions, i.e., $I_{Mie}(q)$ and $I_{internal\ structure}(q)$. Similar results are obtained for the PVA98 solution jet as shown in Figure S5.

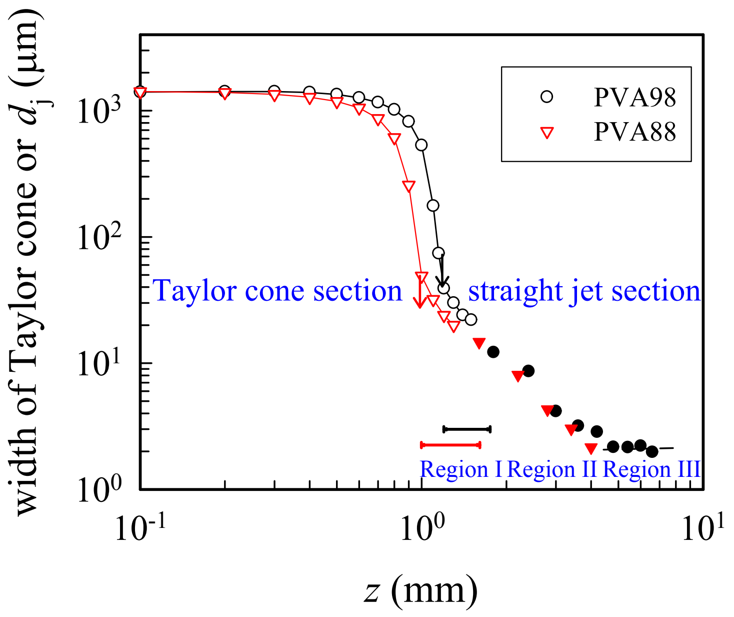

Figure S8. Profiles of the Taylor-cone width and jet diameter $d_j(z)$ as a function of z for the PVA98 solution and PVA88 solution. The width of Taylor cone and the tapering jet around the cone apex (open symbols) is measured from the optical images (Figure S9), while the diameter of straight jet (filled symbols) is determined from the light scattering pattern. The arrow indicates the position of the cone apex for each solution.

In the straight jet section starting from the cone apex until the initiation of jet whipping, three regions are noted; they are denoted as Region I, Region II and Region III[6]. The black bar ranging between the cone apex and the first filled circle symbol indicates Region I for the PVA98 solution, while the red bar shows Region I of the PVA88 solution. In Region II, a power law dependence of d_j on the jet position (z) is evident. In Region III, the jet diameter reaches a constant prior to jet whipping.

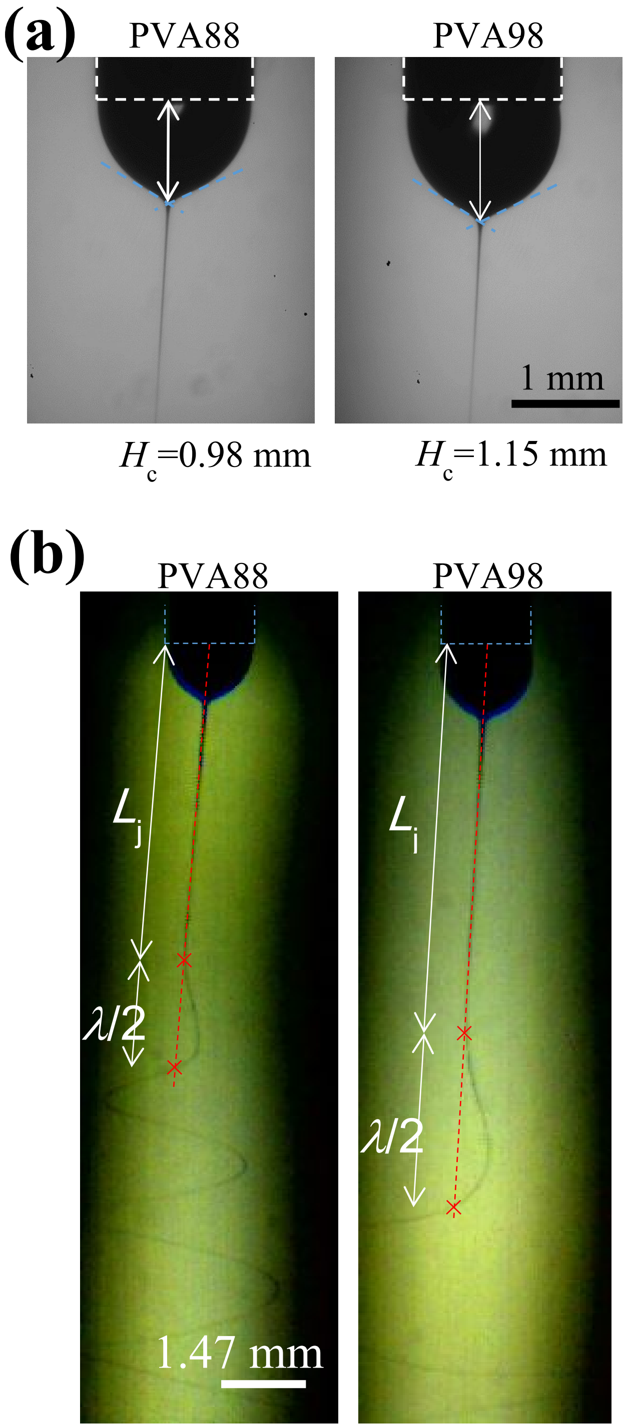

Figure S9. (a) Images of the Taylor cone from which the cone height H_c is measured from the needle tip to the cone apex. (b) images of the whole jet by high-speed video, from which the straight jet length L_j and the half-wavelength $\lambda/2$ of the first jet bend are measured.

(a)

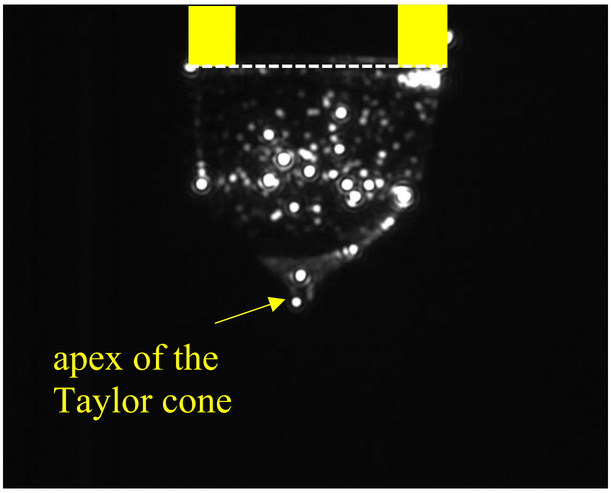

(b)

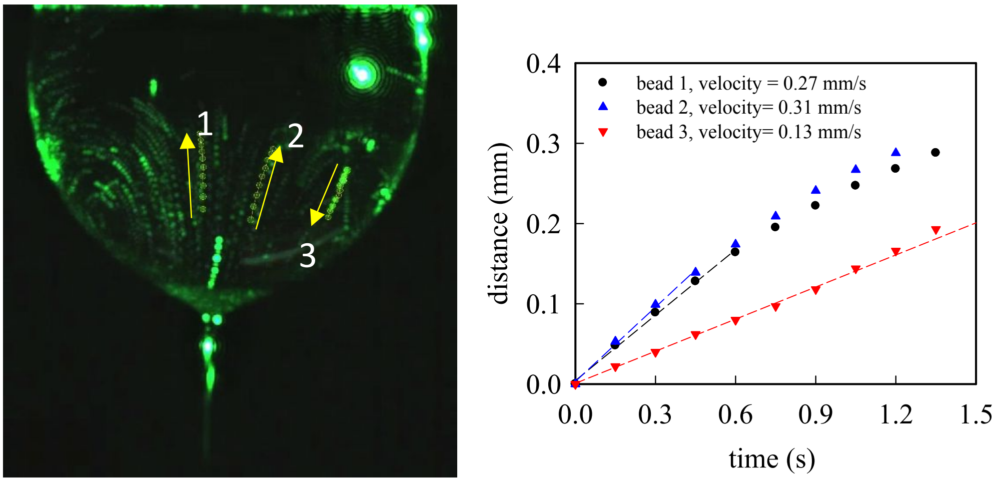

Figure S10. (a) Particle image velocimetry of PVA88/H_2O in the Taylor cone. The yellow parts show the wall of the needle spinneret, and the white line is the needle tip. The stationary white spots are not flowing beads but are the resultant focusing points of the sheet laser light due to the curvatures of the Taylor cone. The details of flow visualization are provided in Movie S1. Similar to the electrospraying of low-viscosity liquids, the presence of toroidal vortex is seen inside the Taylor cone[15]. (b) Determination of the fluid velocity in the Taylor cone by tracing the displacement of beads at various elapsed time. Beads 1 and 2 are for the backflow, whereas bead 3 flows to the cone bottom. The velocity of backflow is gradually reduced since the flowing fluid impinges on the inflow from the spinneret.

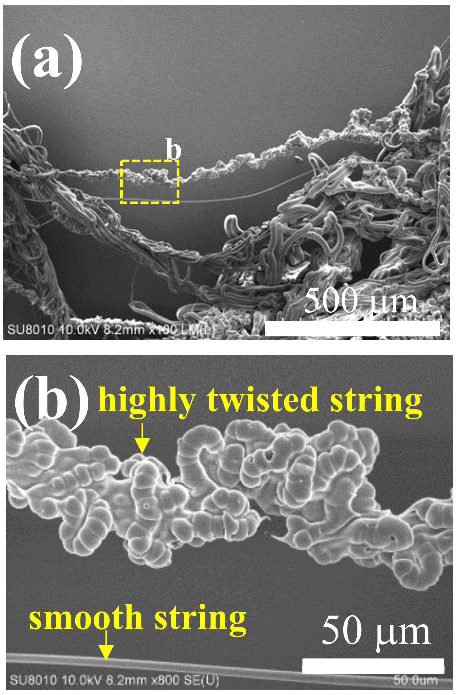

Figure S11. SEM images of the highly twisted jets and strings, which are rapidly frozen by 1-propanol, a nonsolvent. The dashed box in (a) is enlarged to show in (b).

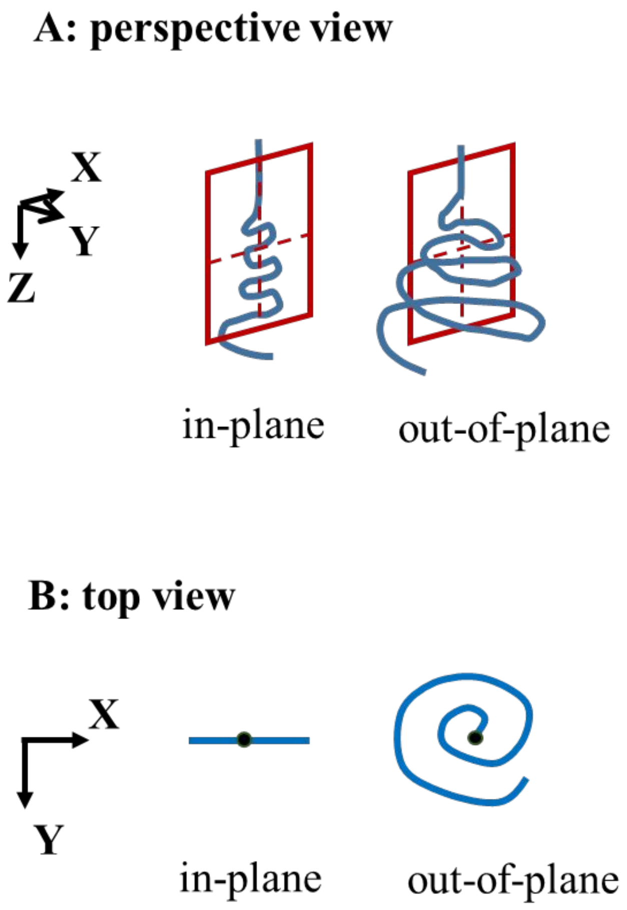

Figure S12. Illustrations of in-plane and out-of-plane lateral vibration of charged jet at the straight jet end. The onset of the jet bend is caused by the air drag to compress the straight jet to induce the jet buckling[6]. If there is no residual torsion at the straight jet end, in-plane lateral vibration of the charged jet occurs. On the other hand, the residual torsion of the jet may produce the spiral motion of the charged jet at the straight jet end; Illustrated is a counterclockwise (LH) spiral jet.

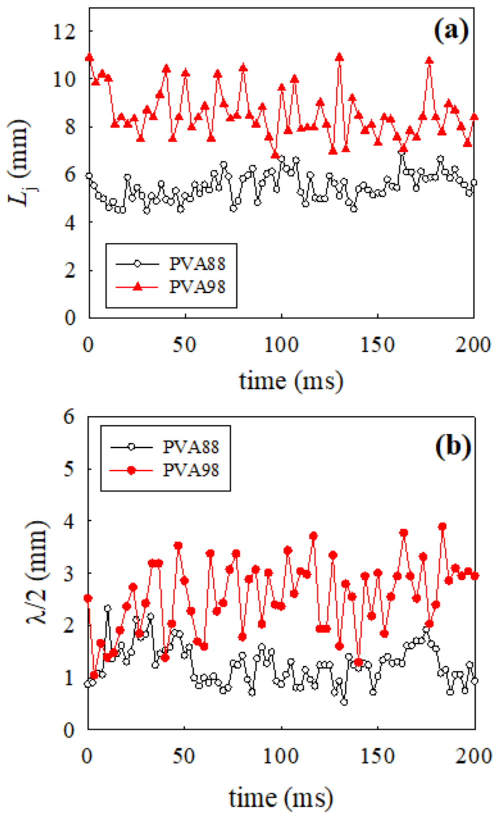

Figure S13. Fluctuations of (a) straight-jet length L_j and (b) the half-wavelength of the spiral jet $\lambda/2$ in a period of 200 ms. The averaged values of L_j and $\lambda/2$ and the corresponding standard deviation are listed in Table 2.

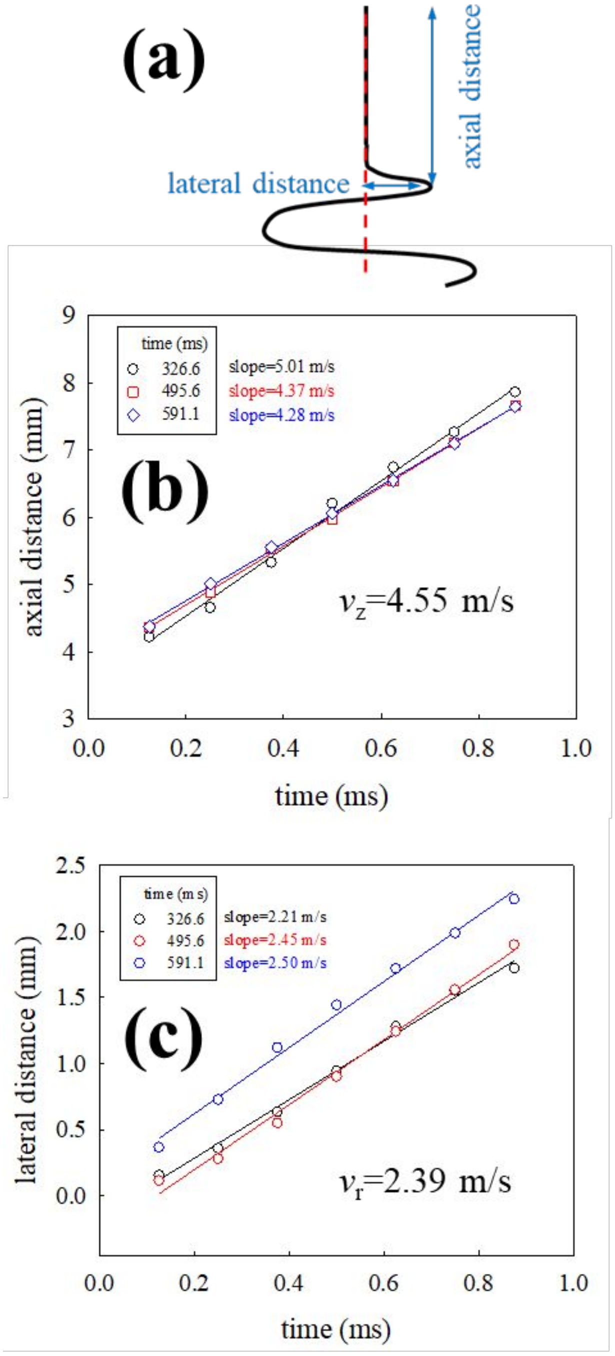

Figure S14. (a) Measurements of the position of the first bend of the spiral jet, i.e., axial distance and lateral distance from the imaginary extension of the straight jet (dashed line), to plot the time dependence of the displacement in (b) and (c) so that the axial velocity (v_z) and lateral velocity (v_r) of the first bend are determined from the slope. [PVA88 solution jet].

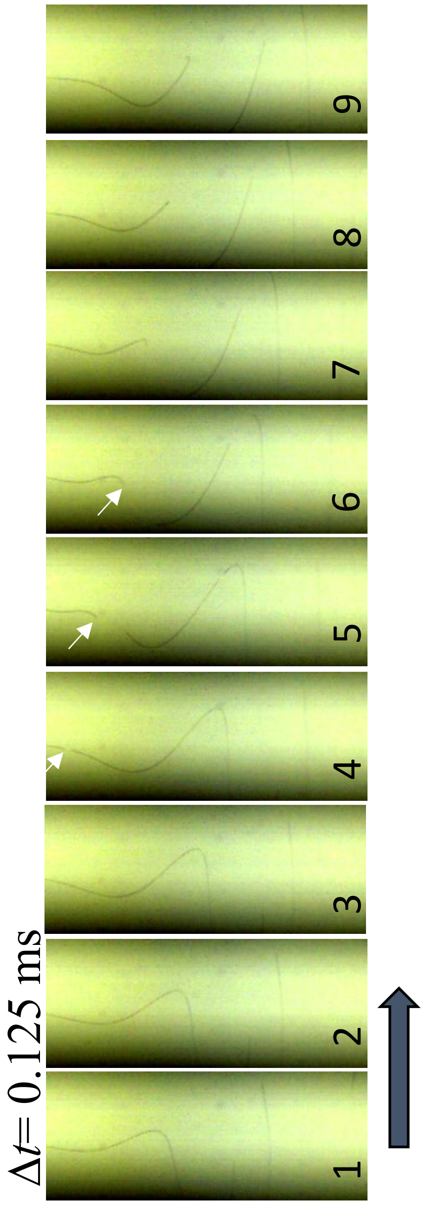

Figure S15. Consecutive snapshots to show the breaking of spiral jet of PVA98 solution at a frame rate of 8000 fps. The white arrow in frame 4 shows the initial breaking point of the spiral jet.
